# Autosomal str allele frequencies, forensic parameters and population structure in four underrepresented indigenous groups from Paraguay

**DOI:** 10.1007/s00414-026-03731-2

**Published:** 2026-03-07

**Authors:** Tamara S. Frontanilla, Karen Nielsen, Katherine Samudio, Romina Arrúa, Juan Garcete, Xavier Ortiz, Renate Henning

**Affiliations:** 1 Ribeirão Preto Medical School, University of São Paulo, Ribeirão Preto, São Paulo Brazil; 2 Díaz Gill Laboratory, Asunción, Paraguay; 3 Paraguayan Institute of Indigenous (INDI), Asunción, Paraguay

**Keywords:** Indigenous populations of Paraguay, Genetic diversity, Short tandem repeats (STRs), Forensic genetics, Population structure

## Abstract

Indigenous populations in Paraguay have historically been excluded from forensic genetic reference databases. In current casework, allele frequency estimates for Indigenous individuals are typically derived from admixed Paraguayan datasets, which may introduce statistical bias and reduce the accuracy of human identification and kinship analyses. Addressing this gap is essential to ensure equitable and appropriate forensic interpretation. Here, we present the first comprehensive autosomal STR reference dataset specifically developed for Indigenous communities in Paraguay. A total of 276 individuals from four distinct groups: Nivaclé (*n* = 119), Enxet Sur/Mbya (*n* = 68), Mbya (*n* = 30), and Aché (*n* = 59) were genotyped for 23 STR loci commonly used in forensic practice. All markers presented high heterozygosity, strong discrimination power, and minimal missing data, supporting their robustness for use in casework and database applications. Population structure analyses indicated that more than 90% of the genetic variation occurs within populations, with only moderate differentiation among them. These findings reinforce the need to treat Indigenous groups as independent reference populations rather than assuming representativeness from admixed national samples. The dataset generated in this study fills a critical gap in South American forensic genetics by incorporating historically underrepresented communities into national reference resources. By improving the accuracy and fairness of match probability estimates, this work contributes to strengthening forensic reliability and promoting a more equitable justice system for Indigenous peoples in Paraguay.

**Supplementary Information:**

The online version contains supplementary material available at 10.1007/s00414-026-03731-2.

## Introduction

Indigenous populations across the Americas represent a valuable component of human genetic diversity, shaped by millennia of migration, adaptation and sociocultural complexity long before European contact [1, 2]. Despite this historical depth, these groups remain largely underrepresented in genomic and forensic research, which limits our ability to make accurate genetic inferences when Indigenous individuals are involved in judicial proceedings.

Paraguay, an inland country in South America, has undergone major demographic transformations that have influenced its present-day genetic landscape. The War of the Triple Alliance (1864–1870) caused one of the most severe population bottlenecks recorded in the region, reducing the national population by 70–85% [3–5]. After the war, the repopulation policies favored intense admixture, leading to homogenization of genetic structure and shaping the predominantly mestizo population observed today [6, 7]. In contrast, Indigenous communities now represent only 2.3% of the national population (~ 140,000 individuals), but retain distinctive genetic and cultural legacies [8]. Guaraní, an Indigenous language, is spoken by over 85% of Paraguayans [9, 10], illustrating a paradox in which Indigenous heritage is central to national identity however, Indigenous peoples themselves remain marginalized in scientific research [11].

The largest ethnic groups include the Mbya Guaraní (20.4%), Ava Guaraní (16.5%), Nivaclé (13.3%), and Paĩ Tavyterã (11.4%), with nearly half of the Indigenous population residing in the Paraguayan Chaco [8]. Given Paraguay’s small population size and the logistical, geographic and legal challenges of fieldwork, our sample of 276 individuals represents a substantial proportion of the national Indigenous population, reinforcing the relevance of this study. In contrast, similar sampling efforts in larger countries typically capture only a small fraction of the target populations.

Scientific research on Paraguayan Indigenous communities remains extremely limited. The first Indigenous census was conducted only in 1981, combined with historical underinvestment in research infrastructure and restricted access to remote communities, this has resulted in these populations being largely absent from the genomic literature [8]. As a result, their genetic, cultural, and social characteristics remain significantly underrepresented in global databases, limiting our understanding of human diversity.

Autosomal short tandem repeats (STRs) remain the primary markers in forensic DNA analysis and provide informative estimates of genetic diversity in both admixed and small populations [12, 13]. Although high-density SNP and sequencing approaches offer deeper resolution, STR-based datasets are essential to establish appropriate forensic parameters where national reference data are incomplete [14–18].

In this context, the present study provides the first population-specific reference dataset for 23 autosomal STR loci in four Indigenous communities from Paraguay. We estimate allele frequencies, evaluate key forensic parameters and assess population structure to determine whether these groups should be treated as independent reference populations in forensic applications. Incorporating these data into forensic practice is expected to improve the accuracy and equity of human identification in cases involving Indigenous individuals.

## Methodology

This study was approved by the Ethics Committee of the National University of Caaguazú (Approval No. CEI° 06/2021). All participants reported being unrelated, and only one family member per household was included to reduce the possibility of cryptic relatedness. Recruitment was conducted in coordination with community leaders, and written informed consent was obtained from all adult participants or legal guardians. Individuals with incomplete consent, sample contamination or insufficient DNA yield were excluded from the analysis. A total of 276 saliva samples were collected from individuals belonging to four Indigenous communities in Paraguay: Nivaclé (*n* = 119), Enxet Sur/Mbya (*n* = 68), Mbya (*n* = 30), and Aché (*n* = 59).

Genomic DNA was extracted using the PrepFiler™ Forensic DNA Extraction Kit [19]. A panel of 23 autosomal STR markers was analyzed: CSF1PO, D1S1656, D2S1338, D2S441, D3S1358, D5S818, D6S1043, D7S820, D8S1179, D10S1248, D12S391, D13S317, D16S539, D18S51, D19S433, D21S11, D22S1045, FGA, PentaE, PentaD, TH01, TPOX, and vWA, following the manufacturer’s instructions. DNA quantification was performed with a NanoDrop 2000 spectrophotometer. Amplified products were run on a SeqStudio Genetic Analyzer, and allele calling was carried out in GeneMapper ID-X v1.6 [20–22]. Quality control procedures followed international forensic guidelines. Each extraction included negative controls, and amplification included positive and negative controls to detect contamination. Allele calls followed ISFG recommendations, and genotypes were accepted only when two consistent reads were obtained. The global genotyping success rate was 100%, with no missing loci across individuals.

Allele frequencies, Hardy–Weinberg equilibrium [23] tests, observed and expected heterozygosities, and forensic parameters (MP, PD, PE and PIC) were calculated for each population and for the combined dataset using GenAlEx v6.5 and STRAF v2.5 [24, 25]. Principal Coordinates Analysis (PCoA) was performed in GenAlEx [23], and population differentiation was evaluated with Analysis of Molecular Variance (AMOVA) using Arlequin v3.5 [26].

To explore broad differentiation patterns using the available STR data, two complementary reference datasets were constructed. Both included the four Indigenous groups and the admixed Paraguayan population previously reported by a previous study [27], but differed in their external reference populations. Dataset 1 incorporated continental populations from the 1000 Genomes Project (15, 28), whereas Dataset 2 included Native American populations from the Human Genome Diversity Project, such as Maya, Pima and Karitiana [29–31]. These datasets were used exclusively as exploratory comparisons to contextualize the Paraguayan samples within broader continental patterns.

Multidimensional Scaling (MDS), Analysis of Molecular Variance (AMOVA), were conducted using STRAF, Arlequin v3.5, and STRUCTURE v2.3.4, respectively [25, 26, 32]. STRUCTURE analyses were performed only as exploratory assessments under the correlated allele frequency model, using a burn-in of 150,000 steps followed by 150,000 MCMC iterations across 100 replicates (K = 2–10), and summarized with CLUMPAK [33]. Given the known limitations of autosomal STRs for ancestry inference, STRUCTURE outputs are presented as supplementary material and interpreted with caution.

## Results

All 276 saliva samples yielded complete genotypes across the 23 STR loci, with no missing alleles, confirming high data quality and the robustness of field and laboratory procedures. Allele frequencies and forensic parameters for the combined Indigenous dataset are presented in Table 1, and population-specific estimates are detailed in the Supplementary Material. Penta E and D18S51 were the most polymorphic markers, and all loci showed high informativeness, with power of discrimination values ranging from 0.702 to 0.966. The cumulative power of discrimination for the 23-locus panel exceeded 0.999999, demonstrating excellent suitability for human identification and kinship analyses in casework involving Indigenous individuals.

**Table 1:** Allele frequencies and forensic parameters in Paraguayan Indigenous populations

Allele/*n*	CSF1PO	D1S1656	D2S1338	D2S441	D3S1358	D5S818	D6S1043	D7S820	D8S1179	D10S1248	D12S391	D13S317	D16S539	D18S51	D19S433	D21S11	D22S1045	FGA	PentaD	PentaE	TH01	TPOX	vWA
**5**																				0,002			
**6**																					0,397		
**7**	0,007					0,098		0,005				0,004								0,007	0,330	0,002	
**8**				0,002		0,004		0,015	0,002			0,024	0,002	0,007					0,040	0,013	0,004	0,457	
**9**				0,002		0,060		0,055	0,004			0,325	0,234						0,116		0,036	0,002	
**9**,**2**																			0,040				
**9**,**3**																					0,234		
**10**	0,230			0,656		0,011	0,002	0,149	0,165			0,049	0,183	0,002			0,004		0,326	0,004		0,007	
**11**	0,230	0,005		0,192	0,002	0,520	0,042	0,409	0,034	0,002		0,136	0,252	0,054	0,002		0,016		0,072	0,034		0,228	
**11**,**3**				0,002																			
**12**	0,411	0,031		0,071		0,248	0,043	0,342	0,230	0,005		0,184	0,179	0,076	0,038				0,158	0,277		0,301	
**12**,**2**															0,069								
**13**	0,112	0,114		0,013		0,058	0,036	0,022	0,275	0,223		0,215	0,132	0,118	0,197				0,132	0,020			
**13**,**2**															0,071								
**13**,**4**																			0,004				
**14**	0,009	0,145		0,060	0,018	0,002	0,353	0,004	0,201	0,469	0,002	0,058	0,011	0,417	0,321		0,007		0,054	0,123			0,031
**14**,**2**															0,007								
**15**		0,111		0,004	0,491		0,016		0,071	0,252	0,009	0,005	0,004	0,120	0,219		0,453		0,056	0,091			0,056
**15**,**2**															0,067								
**15**,**3**		0,009																					
**16**		0,170	0,005		0,250		0,027		0,011	0,045	0,016			0,060	0,009		0,493			0,111			0,382
**16**,**3**		0,063																					
**17**		0,027	0,112		0,217		0,022		0,007	0,004	0,027			0,078			0,027	0,002	0,002	0,096			0,321
**17**,**3**		0,264									0,002												
**18**		0,002	0,248		0,018		0,067				0,159			0,051				0,011		0,082		0,004	0,181
**18**,**3**		0,045									0,009												
**19**			0,089		0,004		0,156				0,332		0,004	0,002				0,040		0,058			0,024
**19**,**3**		0,013																					
**20**			0,116								0,337			0,005				0,011		0,045			0,005
**20**,**3**							0,069																
**21**			0,002								0,071			0,005				0,091		0,024			
**21**,**3**							0,165																
**22**			0,065								0,011							0,069		0,011			
**22**,**3**							0,002																
**23**			0,315								0,024							0,207		0,004			
**23**,**2**																		0,014					
**24**			0,042											0,005				0,134					
**24**,**2**																		0,013					
**24**,**3**											0,002												
**25**			0,005															0,295					
**26**																		0,096					
**27**																0,002		0,018					
**28**																0,004							
**29**																0,138							
**30**																0,295							
**30**,**2**																0,011							
**31**																0,020							
**31**,**2**																0,145							
**32**,**2**																0,293							
**32**,**3**																0,002							
**33**,**2**																0,083							
**33**,**3**																0,004							
**35**																0,004							
**N**	276	276	276	276	276	276	276	275	276	276	276	275	276	276	276	276	276	276	276	276	276	276	276
**Na**	6	13	10	9	7	8	13	8	10	7	13	9	9	14	10	12	6	13	11	17	5	7	7
**Ho**	0,670	0,772	0,699	0,514	0,605	0,630	0,764	0,687	0,746	0,591	0,750	0,749	0,750	0,692	0,761	0,754	0,529	0,772	0,804	0,826	0,670	0,652	0,678
**He**	0,712	0,847	0,799	0,524	0,649	0,651	0,808	0,690	0,797	0,665	0,744	0,789	0,799	0,777	0,794	0,779	0,551	0,827	0,823	0,864	0,678	0,649	0,714
**MP**	0,131	0,042	0,071	0,270	0,174	0,162	0,055	0,157	0,072	0,164	0,108	0,074	0,074	0,080	0,075	0,085	0,298	0,051	0,052	0,034	0,168	0,200	0,137
**PE**	0,384	0,548	0,427	0,200	0,297	0,329	0,535	0,409	0,504	0,280	0,510	0,508	0,510	0,416	0,529	0,516	0,214	0,548	0,607	0,648	0,384	0,358	0,394
**PD**	0,869	0,958	0,929	0,730	0,826	0,838	0,945	0,843	0,928	0,836	0,892	0,926	0,926	0,920	0,925	0,915	0,702	0,949	0,948	0,966	0,832	0,800	0,863
**PIC**	0,664	0,830	0,773	0,484	0,589	0,607	0,788	0,636	0,767	0,607	0,705	0,760	0,768	0,758	0,767	0,746	0,450	0,808	0,805	0,852	0,614	0,580	0,665

Hardy–Weinberg equilibrium deviations were limited to isolated locus-population combinations, with no systematic departures observed, suggesting that potential deviations are likely due to localized demographic patterns rather than consistent inbreeding.

**Fig. 1:**
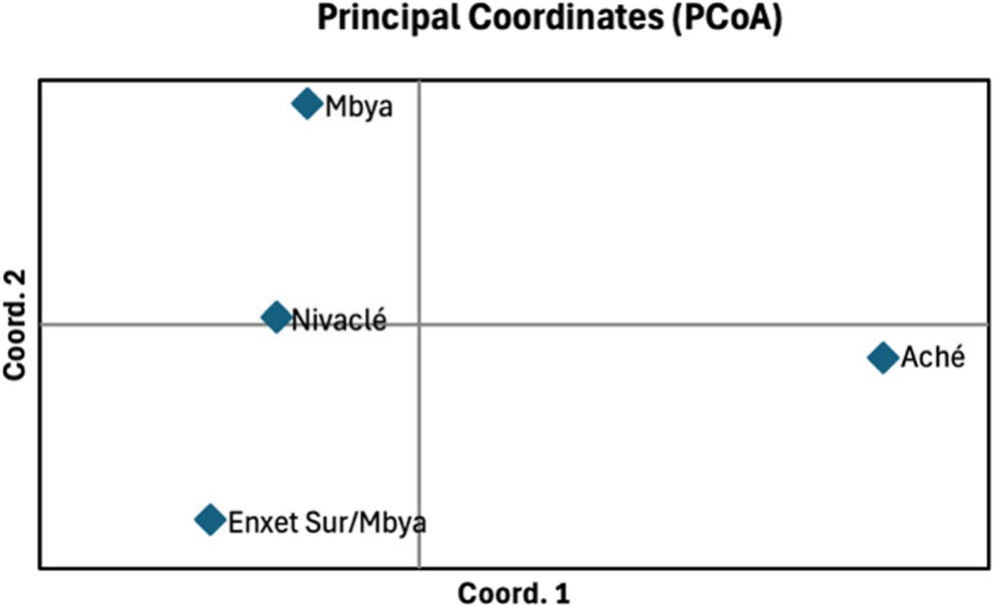
Principal coordinates analysis for the Paraguayan indigenous populations

Population structure analyses revealed clear genetic differentiation among the four Indigenous groups. Principal Coordinates Analysis (PCoA) indicated visually distinct clustering (Fig. 1), where the Nivaclé individuals occupied an intermediate position, Mbya and Aché appeared at opposite extremes of the axes, and Enxet Sur/Mbya showed partial separation consistent with sampling locality. AMOVA supported these results, with 7.43% of the total variance attributable to differences among populations and 92.57% within individuals.

**Fig. 2:**
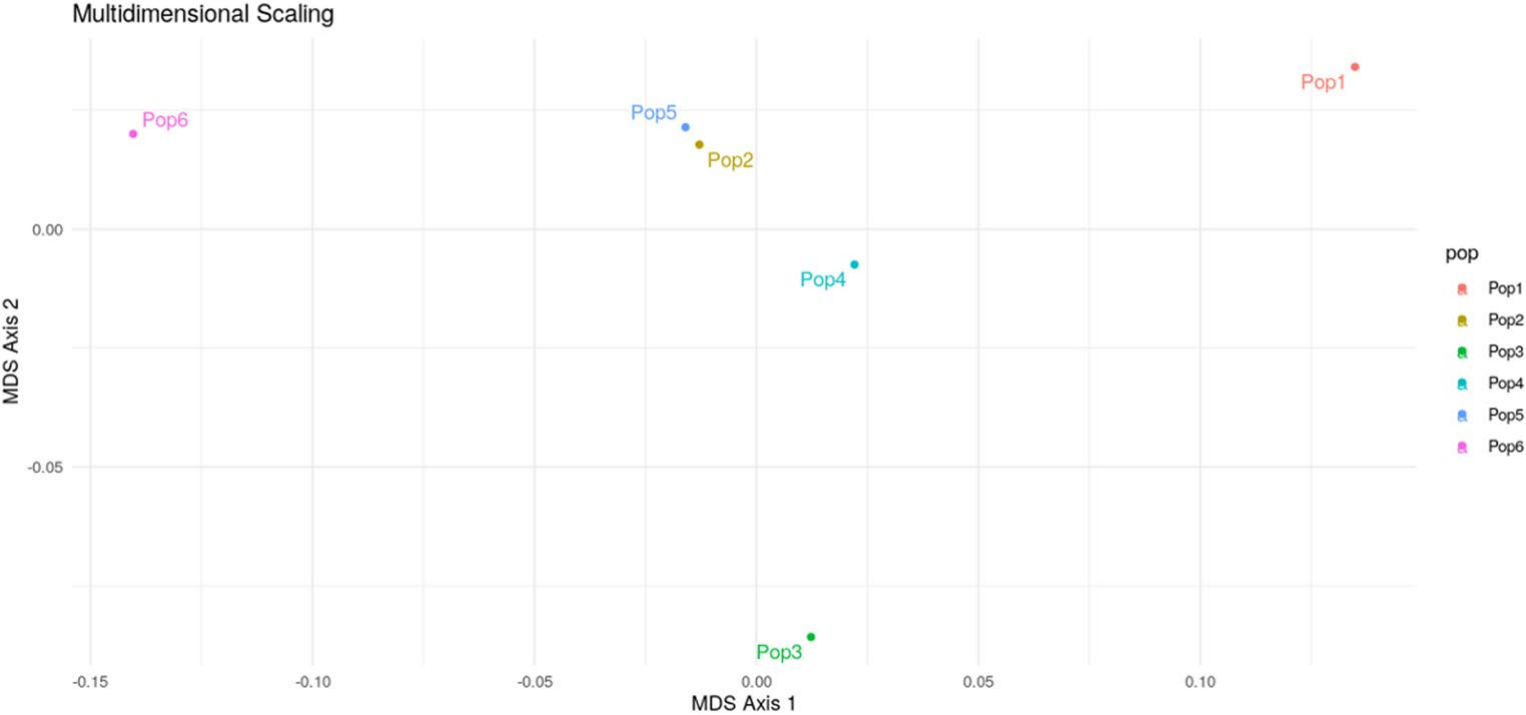
Multidimensional Scaling using the first dataset (1000 Genomes Project). Pop 1: African; Pop 2: American; Pop 3: East Asia; Pop 4: Europe; Pop 5: Paraguay; Pop 6: Paraguayan Indigenous

To contextualize these patterns within broader geographic scales, an exploratory Multidimensional Scaling (MDS) analysis including continental populations from the 1000 Genomes Project showed the Indigenous Paraguayans forming a compact cluster clearly separated from African, East Asian and European groups (Fig. 2), while the admixed Paraguayan population clustered closer to other American references. A second exploratory MDS with Native American groups from the HGDP dataset produced similar trends (Supplementary Fig. 1), indicating regional differentiation within South America. AMOVA for these broader comparisons again confirmed that > 96% of total variation resided within populations.

An exploratory Bayesian clustering analysis (STRUCTURE) was conducted to complement multivariate patterns. As expected for autosomal STR datasets, clustering captured major continental separations at low K values, while higher K values yielded diffuse profiles with limited resolution (Supplementary Fig. 2). These results are presented solely in the Supplementary Material and were not used for ancestry inference. All four Indigenous groups showed detectable but moderate differentiation, as well as high intrapopulation diversity and robust forensic performance across all loci.

## Discussion

This study provides the first population-specific autosomal STR database for Indigenous groups in Paraguay, addressing a major gap in national forensic resources and strengthening the reliability of human identification in cases involving these communities [27]. In current practice, match probabilities for Indigenous individuals are typically estimated using allele frequencies from admixed Paraguayans, which may lead to inaccurate likelihood estimates and reduced statistical power in legal proceedings [34, 35]. The dataset presented here enables more appropriate population assignment and contributes to improving equity in forensic interpretation.

The high heterozygosity observed across loci demonstrates that autosomal STRs remain highly informative markers in small and underserved populations with limited access to genomic resources [36]. Comparable levels of polymorphism have been described in Indigenous groups from Brazil, Bolivia and Argentina [37–39], supporting the continued relevance of STR-based approaches for diversity studies and casework applications across South America. Population structure analyses revealed detectable yet moderate differentiation among the four Indigenous groups. Most genetic variation (> 92%) was attributable to differences within populations, a pattern reported in other Native American communities where geographic and cultural boundaries generate differentiation without reducing internal diversity [2, 40]. Localized deviations from Hardy–Weinberg expectations are consistent with demographic drift and specific histories rather than systematic inbreeding, reflecting traditional exchange practices documented in Guaraní and Amazonian societies [41, 42].

Exploratory comparisons with external reference datasets were used only to contextualize the Paraguayan groups within continental patterns. Indigenous Paraguayans remained genetically distinct from the admixed national population, consistent with historical and sociocultural limits to recent gene flow [7], and positioned near other Native American groups, as expected for a shared ancestral origin followed by regional diversification across the continent [2, 43]. Importantly, because autosomal STRs have limited resolution for ancestry inference, these results should not be interpreted as quantitative estimates of admixture or demographic history.

The observed affinity between admixed Paraguayans and European populations reflects the known consequences of the War of the Triple Alliance and subsequent repopulation processes dominated by European paternal contributions [4, 44]. These findings support the need to treat Indigenous and admixed Paraguayans as genetically distinct groups in forensic statistical calculations rather than using a single national frequency database.

Finally, this work contributes to reducing the structural invisibility of Indigenous peoples in genomic resources. Persistent barriers such as geographic isolation, language differences and administrative constraints continue to limit the representation of these communities in scientific research. Expanding forensic databases to include Indigenous populations is therefore essential not only for analytical accuracy but also for ensuring fair access to justice.

## Limitations and future perspectives

Although based on a widely used forensic panel, STR markers provide only moderate resolution of population structure. Incorporation of high-density SNP data and sequencing STR profiling will allow deeper characterization of internal diversity and admixture dynamics in future studies. Increasing sample sizes and including additional communities will further strengthen the forensic value and representativeness of Indigenous datasets in Paraguay.

## Conclusion

This study provides the first population-specific autosomal STR reference dataset for Indigenous communities in Paraguay, enabling more accurate and equitable statistical interpretation in forensic casework involving these groups. The 23 locus panel showed high forensic performance and robust intrapopulation diversity, supporting its inclusion as a dedicated resource in national databases. Although detectable differentiation exists among Indigenous groups, most genetic variation occurs within populations, reinforcing the need to avoid using admixed Paraguayan frequencies as a default. Increasing Indigenous representation in forensic genetics improves the accuracy of human identification in South America and represents a necessary step towards more inclusive justice systems. Future efforts will incorporate genome markers and additional communities looking to improve the resolution and practical applicability of these reference data.

## Supplementary Information

Below is the link to the electronic supplementary material.

Supplementary Material 1 (JPG 42.9 KB)

Supplementary Material 2 (JPG 404 KB)

Supplementary Material 3 (DOCX 14.6 KB)
