# Supplementary figures and images for: Autosomal str allele frequencies, forensic parameters and population structure in four underrepresented indigenous groups from Paraguay
Source: Int J Legal Med. 2026 Mar 7;140(4):1943–50. doi: 10.1007/s00414-026-03731-2 (PMC13275611; doi:10.1007/s00414-026-03731-2)

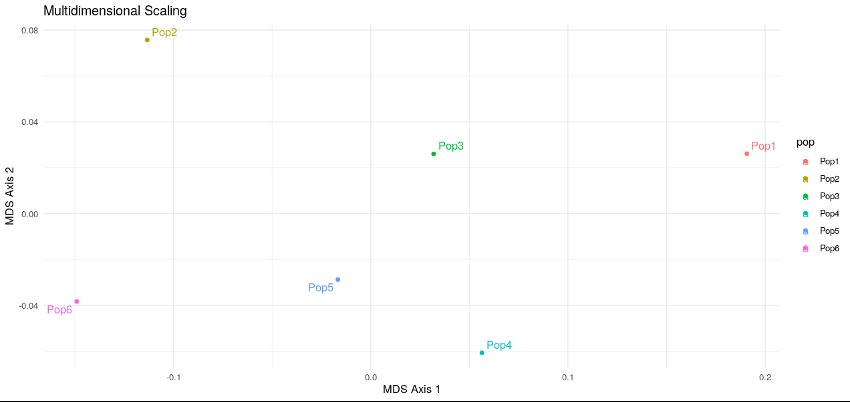

Supplement: Supplementary file 1 — Supplementary Material 1 (JPG 42.9 KB) [file 414_2026_3731_MOESM1_ESM.jpg]

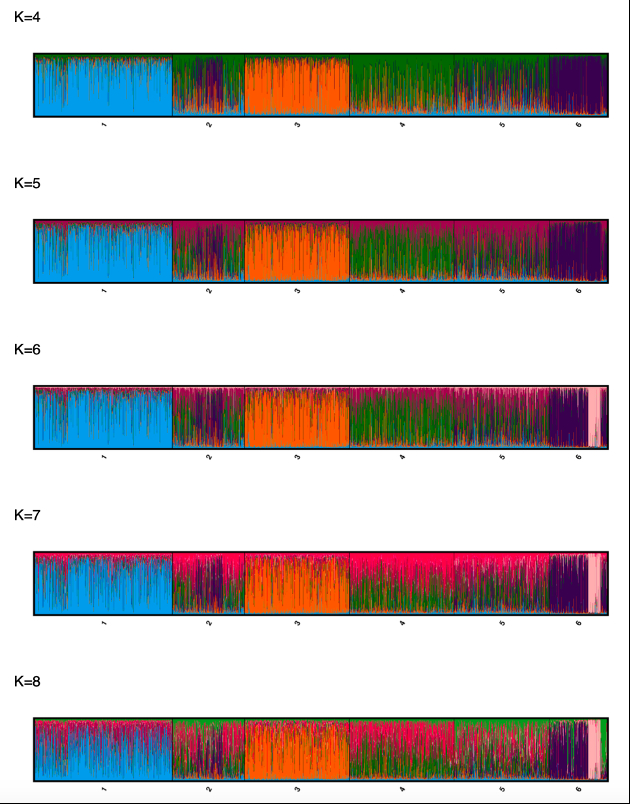

Supplement: Supplementary file 2 — Supplementary Material 2 (JPG 404 KB) [file 414_2026_3731_MOESM2_ESM.jpg]
