# Supplementary material for: Autosomal str allele frequencies, forensic parameters and population structure in four underrepresented indigenous groups from Paraguay
Source: Int J Legal Med. 2026 Mar 7;140(4):1943–50. doi: 10.1007/s00414-026-03731-2 (PMC13275611; doi:10.1007/s00414-026-03731-2)
Supplement: Supplementary file 3 — Supplementary Material 3 (DOCX 14.6 KB) [file 414_2026_3731_MOESM3_ESM.docx]

**Supplementary information**

**Supplementary Fig. 1.** Multidimensional Scaling (MDS) based on allele frequency distances using 23 autosomal STR markers. The plot includes the four Indigenous groups analyzed in this study, the admixed Paraguayan population, and Native American reference groups (Maya, Pima and Karitiana) from the Human Genome Diversity Project (HGDP). Each point represents a population; colors indicate group identity as shown in the legend. Axes correspond to the first two MDS dimensions. This analysis is provided as an exploratory comparison to illustrate relative genetic affinities and should not be interpreted as formal ancestry inference due to the limited resolution of STR markers.

**Supplementary Fig. 2.** Bayesian clustering results (STRUCTURE) based on 23 autosomal STR loci. Each vertical bar represents an individual, and colors denote inferred ancestry components (clusters). Populations are shown in separate blocks: (1) African, (2) American, (3) East Asian and (4) European groups from the 1000 Genomes Project, (5) admixed Paraguayans, and (6) the four Indigenous communities from Paraguay. Results are displayed for K = 4 to K = 8. For each value of K, the plot corresponding to the run with the highest log-likelihood was selected and graphically aligned using CLUMPAK. This analysis is presented as an exploratory assessment to illustrate broad patterns of genetic affinity and should not be interpreted as quantitative ancestry inference, given the limited resolution of STR data.
